# Influence of Bioceramic Cements on the Quality of Obturation of the Immature Tooth: An In Vitro Microscopic and Tomographic Study

**DOI:** 10.3390/bioengineering11030213

**Published:** 2024-02-23

**Authors:** Raya Al-Rayesse, Ossama Al-Jabban, Ammar Eid, Alaa Kabtoleh, Frédéric Addiego, Davide Mancino, Youssef Haikel, Naji Kharouf

**Affiliations:** 1 Department of Endodontics and Restorative Dentistry, Damascus University, Damascus 011, Syrian Arab Republic; raya.alrayes@damascusuniversity.edu.sy (R.A.-R.); ossama.jabban@damascusuniversity.edu.sy (O.A.-J.); alaa.kabtoleh@gmail.com (A.K.); 2 Department of Endodontics and Operative Dentistry, Faculty of Dentistry, International University for Science, Damascus 011, Syrian Arab Republic; ammarendo89@gmail.com; 3 Materials Research and Technology (MRT) Department, Luxembourg Institute of Science and Technology (LIST), L-4362 Esch-sur-Alzette, Luxembourg; frederic.addiego@list.lu; 4 Department of Biomaterials and Bioengineering, INSERM UMR_S 1121, Strasbourg University, 67000 Strasbourg, France; mancino@unistra.fr (D.M.); youssef.haikel@unistra.fr (Y.H.); 5 Department of Endodontics, Faculty of Dental Medicine, Strasbourg University, 67000 Strasbourg, France; 6 Pôle de Médecine et Chirurgie Bucco-Dentaire, Hôpital Civil, Hôpitaux Universitaire de Strasbourg, 67000 Strasbourg, France

**Keywords:** calcium silicate, calcium aluminosilicate, premixed cement, powder–liquid cement, open apex

## Abstract

The present in vitro study focuses on the filling ability of three different bioceramic cements with or without the addition of a bioceramic sealer in an open apex model on the marginal apical adaptation, tubule infiltrations, and void distributions as well as the interface between the cement and the sealer materials. To this end, sixty mandibular premolars were used. MTA-Biorep (BR), Biodentine (BD), and Well-Root Putty (WR) were used to obturate the open apex model with or without the addition of a bioceramic sealer, namely TotalFill^®^ BC sealer™ (TF). A digital optical microscope and scanning electron microscope (SEM) were used to investigate the cement–dentin interface, marginal apical adaptation, and the material infiltration into the dentinal tubules. Micro-computed X-ray tomography and digital optical microscopy were used to investigate the cement–sealer interface. The results were analyzed by using the Kruskal–Wallis test. No significant difference was found between the groups for the marginal apical adaptation quality (*p* > 0.05). Good adaptation of the dentin–cement interface was found for all tested groups and the sealer was placed between the cement material and dentinal walls. All the groups demonstrated some infiltrations into the dentinal tubules at the coronal part except for the BR group. A good internal interface was found between the cement and the sealer with the presence of voids at the external interface. A larger number of voids were found in the case of the BD-TF group compared to each of the other two groups (*p* < 0.05). Within the limitations of the present in vitro study, all the groups demonstrated good marginal apical adaptation. The use of a sealer in an open apex does not guarantee good filling and, in addition, creates voids at the external interfaces with the dental walls when the premixed sealer is used with powder–liquid cement systems. The use of a premixed bioceramic cement could offer fewer complications than when a powder–liquid cement system is used.

## 1. Introduction

Incomplete root development is one of the most frequent complications observed in traumatized teeth when the vitality of the pulp vanishes before the accomplishment of dentin deposition [1]. Consequently, the probability of fracture could be increased due to the thin and weak dentinal walls of the root [2]. Regenerative endodontic treatment has gained significant interest in the endodontic domain. This treatment could affirm the replacement of the damaged dental structure [1]. However, this treatment could not reach its purpose, especially in immature permanent teeth with apical periodontitis, pulpal necrosis, or roots with previous unsuccessful endodontic treatments [2,3]. Therefore, apexification using an apical plug or long-term calcium hydroxide application could be the best choice to ensure the healing of apical pathosis and achieve apical closure [4].

Open apex treatment in endodontics is a challenging procedure due to the anatomic configuration of the apex, the difficulty of the delivery of the endodontic material to the apical third without over- or short filling, and the ability of 3D obturation to fill the root canal with minimal voids [5]. This is a nonsurgical procedure that is used to treat immature or incomplete root canal development [5,6].

Historically, calcium hydroxide was used during several visits to induce a barrier of hard tissue before the obturation of the open apex [5]. Recently, several studies showed that treatment of the open apex with calcium silicate materials could improve the efficiency of this treatment within one visit [7,8,9]. These materials showed their efficiency in different endodontic treatments such as apicoectomy [10], perforation [11], pulpotomy [12], open apex [5], pulp cupping [13], and resorption [14]. These calcium-silicate-based materials, called bioceramics, have excellent physicochemical, mechanical, and biological properties [15]. In addition, these materials are commercialized as sealers (liquid) and cements (putty; “thicker than the sealer”) [16].

Mineral trioxide aggregate, “MTA”, was introduced in 1993 and considered the gold standard material for several endodontic treatments [17]. Different calcium silicate cements were developed to improve the quality of the initial MTA. They replaced bismuth oxide, which is toxic and could change the color of the teeth, with calcium tungstate, which is more biocompatible as well as allows the addition of organic plasticizers to the liquid of this cement [15]. MTA Biorep (Itena Clinical, Paris, France) is a powder–liquid calcium silicate cement that is used on endodontic treatment and showed its efficiency among in vitro and in vivo studies [18,19]. Another commercial product that is used worldwide is Biodentine (Septodont, Saint-Maur-des-Fossés, France). This product is a calcium silicate material that is commercialized in powder–liquid formulations with an advantage that its mechanical properties are close to those of dentine [20].

Ashi et al. [19] and Kharouf et al. [21] demonstrated that the premixed bioceramic materials among sealer or cement formulations are better than the bioceramics in powder–liquid formulations in terms of their filling ability, application, and handling. Novel premixed bioceramic materials based on calcium aluminosilicate have recently been introduced in the dental market, an example being Well-Root™ PT (Vericom, Chuncheon-si, Republic of Korea) [21]. It has been reported that the physicochemical, biological, and mechanical properties of this material are comparable to those of MTA and Biodentine™ [21,22,23]. There are no studies in the literature of the three mentioned bioceramic cements and a calcium silicate sealer in the obturation step. Moreover, the effect of using a calcium silicate sealer combined with a putty form has rarely been studied. 

The purpose of the present in vitro study was to investigate the filling ability of each of the three different bioceramic cements with or without the addition of a bioceramic sealer in an open apex model using different imaging techniques as well as investigation of the cement–sealer interfaces. The null hypotheses were that there was no difference between the three bioceramic cements with respect to marginal apical adaptation, the state of the cement–dentin interface, the extent of infiltration of tubules, and the state of the cement–sealer interface (when a sealer is used as a supplementary material) on the quality of obturation of a single-rooted canal in an open apex model. The cements used in the present study have different formulations (powder–liquid or premixed) and chemical compositions (tricalcium silicate or calcium aluminosilicate); thus, several studies mentioned that these different characteristics could play an important role on the biological, physicochemical, and mechanical properties [15,19,21].

## 2. Materials and Methods

### 2.1. Materials

Biodentine^TM^ “BD” (Septodont, Saint-Maur-des-Fossés, France) and MTA Biorep “BR” (Itena Clinical, Paris, France) as powder–liquid cements, and Well-Root PT “WR” (Vericom, Gangwon-do, Republic of Korea) as a premixed cement were used in the present study following their manufacturer instructions (Table 1). TotalFill^®^ BC Sealer™ “TF” (FKG, La Chaux-de-Fonds, Switzerland) was used in the supplementary obturation step.

### 2.2. Teeth Preparation

Sixty freshly extracted first mandibular premolars, with a single canal and root curvature <10° extracted for orthodontic or surgical reasons from patients aged between 18–25 years, were obtained under patient informed consent after obtaining approval from the Ethics Committee at Damascus University, Syria (protocol no. 2571/2023). The recruited teeth were stocked in saline solution at 4 °C until their use [21]. The cusps of the teeth were flattened using a rotating polishing machine (Escil, Chassieu, France) with carbide grit paper (600 grit) to have a standard root canal length of 18 ± 1 mm. A single operator (endodontist: R.A.) performed all the clinical steps. The access cavity was prepared under an optical microscope (Zumax Medical Co., Ltd., New District Suzhou, Jiangsu, China) by using burs and ultrasonic tips. A #10 K-file (Coltene, Lezennes, France) was used to perform the canal scouting step. Then, 0.06 tapered rotary files (Eighteeth, Changzhou City, Jiangsu Province, China) were used by orthograde direction to a master apical file of #30. The open apex divergent was created by using 0.06 tapered rotary files (Eigthteeth, Changzhou City, Jiangsu Province, China) in a retrograde direction to a master file of #30. The diameter of the anatomic open apex was 1.26 mm after the use of #30/0.06 in a retrograde direction to its total length. Then, a final irrigation protocol was used for all the specimens as follows: 5 mL of 17% EDTA solution (2 min), 2.5 mL of 0.9% NaCl (1.5 min), 5 mL of a 6% NaOCl (2 min), followed by a final rinsing with 2.5 mL of 0.9% NaCl (1.5 min). A 31-gauge Navitip needle with a 5 mL syringe was used to deliver all the irrigants by orthograde direction. Teeth were randomized using a computer-generated random sequence at www.randomizer.org and divided into 6 equal groups (*n* = 10) according to the following classifications: 

Group 1 (G1): BD: After mixing, the cement was applied using MapOne carrier (MapOne system, Produits Dentaires SA, Verey, Switzerland);

Group 2 (G2): BD-TF: TF was applied by using a gutta percha point with a painting technique; after that, the cement was applied using MapOne carrier;

Group 3 (G3): BR: After mixing, the cement was applied using MapOne carrier;

Group 4 (G4): BR-TF: TF was applied by using a gutta percha point with a painting technique; after that, the cement was applied using MapOne carrier;

Group 5 (G5): WR: The premixed cement was applied using MapOne carrier;

Group 6 (G6): WR-TF: TF was applied by using a gutta percha point with a painting technique; after that, the cement was applied using MapOne carrier.

Before plug placement, the teeth were embedded in a moistened floral sponge [26]. After drying the canal with paper points, the same operator performed the placement of apical plug in an orthograde direction to rich a 6 ± 0.5 mm plug length coronal to the apex. The plugs were condensed by using fitted pluggers (Dentsply Sirona, Germany). The operator used an operating microscope (Zumax Medical Co., Ltd., New District Suzhou, Jiangsu, China) during the placement of cements. Before injecting warm gutta percha to fill up the root canal using the Fast-pack Pro system (Eighteeth, Hangzhou City, Jiangsu Province, China); a final radiograph (buccal–lingual direction) was taken for each filled tooth to verify the plug length. All the specimens were conserved in the dark in a container at 37 °C and 95% relative humidity for 48 h until completely set [27].

### 2.3. Apical Region Evaluation

The quality of obturation in the apical region of each root was investigated using a digital optical microscope (Keyence, Osaka, Japan) at 50× and 100× magnifications. Micrographs of the filled root apices were evaluated by 2 blinded endodontists. Grading criteria were used to evaluate the marginal adaptation based on a previous study [5]:

Score 1: Root-filling material is well adapted: close marginal approximation of the filling material to the dentinal wall. No spacing defect present at the material–dentin interface in >70% of the circumference of the open apex;

Score 2: Root-filling material is moderately adapted: close marginal approximation of the filling material to the dentinal wall. Presence of spacing defects at the material–dentin interface in 30–60% of the circumference of the open apex;

Score 3: Root-filling material is poorly adapted: poor marginal approximation of the filling material to the dentinal wall. Major voids and/or significant spacing defects at the material–dentin interface in > 60% of the circumference of the open apex.

When different scores were attributed by the two examiners, they reanalyzed the micrograph with a third endodontist to reach an agreement.

### 2.4. Cement–Dentin Interface Investigation

To evaluate the interface adaptation among the different groups, all the teeth were sectioned longitudinally down the middle in the buccal–lingual direction by using a precision cutting machine (Micracut 152, Metkom, Bursa, Turkey) under continuous water cooling. The quality of the interface was investigated using a digital optical microscope at 50× magnification. A qualitative evaluation was performed by the same examiners.

After that, 1200, 2400, and 4000 P-grade (number of abrasive grains per cm^2^) abrasive papers were used to polish the longitudinal section surfaces. Then, the surfaces were treated with 37% phosphoric acid for 10 s and 2.5% NaOCl for 3 min to eliminate the smear layer that was created during sectioning. They were then mounted on SEM stubs and sputter-coated with gold–palladium (20/80). The samples were analyzed using a scanning electron microscope (SEM) (FEI Company, Eindhoven, The Netherlands) at 10 kV and magnifications of 50×, 200× and 1000× with a working distance of 10 mm. SEM imaging was focused on the cement–dentin interfaces along the plug length as well as the cement or sealer material infiltrations into the dentinal tubules.

### 2.5. Cement–Sealer Interface Investigation

**Figure 1:**
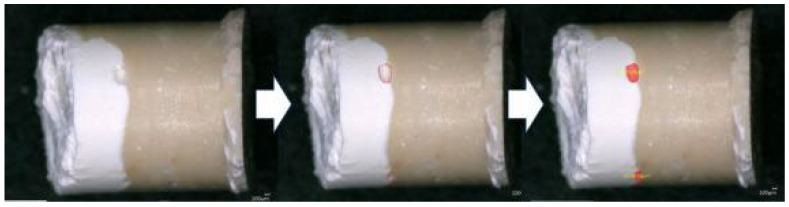
Digital micrographs (BD-TF) with the void quantification by using the software VHX-5000.

Four cylinders from each cement–sealer group were prepared by using a Teflon mold (height: 3.8 mm; diameter: 3 mm). Each cement was firstly placed in the mold to achieve around 2 mm of height; then, the sealer was injected onto the cement material to fill the mold’s remaining space. All the samples were incubated at 37 °C for 48 h to allow the materials to set properly. After the storage period, the samples were removed from the mold and observed on two sides (2 images for each cylinder) using a digital optical microscope to check the cement–sealer interfaces at 70× magnification. Then, the external voids localized at each cement–sealer interface were measured by using VHX-5000 software (Keyence, Osaka, Japan) (Figure 1).

After the external interface observations were made using a digital optical microscope, and due to the difficulty of the investigation of the interface in the WR-TF group, 3D imaging by means of micro-computed X-ray tomography (EasyTom 160 from RX Solutions, Chavanod, France) was performed. This analysis was focused on the internal and external cement–sealer interfaces in the case of one sample per group. The projections were recorded at a voltage of 45 kV, a current of 160 µA, and a frame rate of 0.5 image/s. The source-to-detector distance (SDD) was set to about 293.6 mm, whereas the source-to-object distance (SOD) was set at about 5.6 mm, providing a voxel size of about 2.5 µm. A total of 1440 projections were obtained over 360° with a total acquisition time of 48 min. The volume reconstruction was performed with the software Xact64 (RX Solutions) and the 3D image analysis was performed using the Avizo software (ThermoFisher, Waltham, MA, USA).

**Figure 2:**
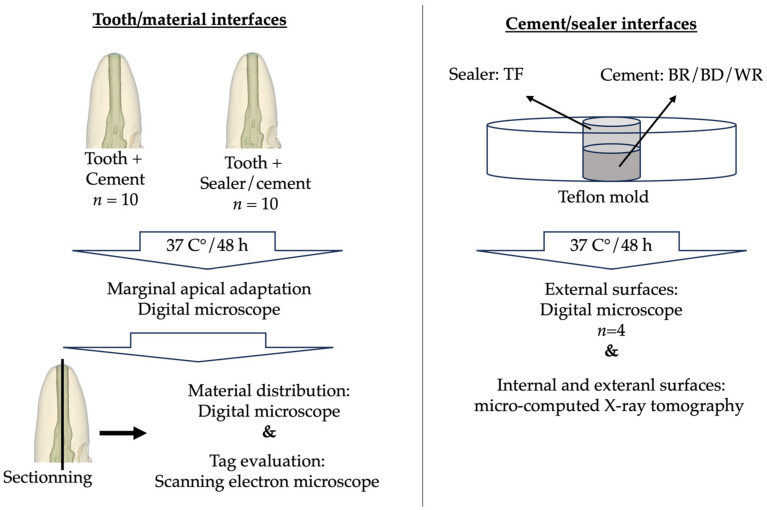
Sample preparation and investigation methods.

Figure 2 shows the sample preparation protocols and the different investigation methods.

### 2.6. Statistical Analysis

SigmaPlot release 11.2 (Systat Software, Inc., San Jose, CA, USA) was used to analyze the results. To determine the significance of the difference in the marginal apical adaptation, tag infiltration length at the cement–dentin interface, and void measure (area and volume) at the cement–sealer interface (in groups where a sealer was used), the Kruskal–Wallis test including comparison procedures (Tukey’s Test) was used. A significant difference was indicated when *p* < 0.05.

## 3. Results

### 3.1. Marginal Apical Adaptation

**Figure 3:**
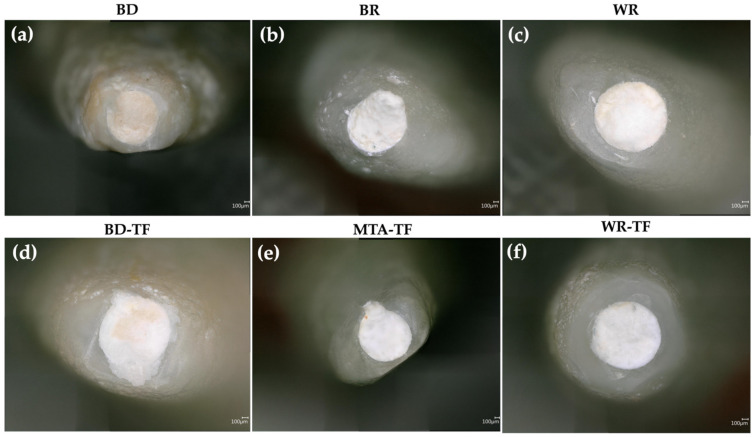
Digital micrographs highlighting the marginal apical adaptation in (**a**–**c**) teeth filled with the different cements and (**d**–**f**) teeth filled with sealer–cements. Biodentine: BD; BR: MTA Biorep; Well-Root PT: WR; Biodentine + Total Fill: BD-TF; MTA Biorep + Total Fill: BR-TF; Well-Root PT+ Total Fill: WR-TF.

Micrographs showing the marginal apical adaptations are presented in Figure 3.

After the evaluation of the different micrographs by the examiners, there was no statistically significant difference in the marginal adaptation between the tested groups (*p* > 0.05) (Table 2). Moreover, the use of a sealer for painting the dentinal walls before the obturation with the cements produced no significance difference in the quality of marginal adaptation for G1 vs. G2 (*p* = 0.2); G3 vs. G4 (*p* = 0.06) and G5 vs. G6 (*p* = 0.146). In addition, no statistically significant difference was detected between the groups of cements (*p* = 0.576) as well as between the groups of sealer/cements (*p* = 0.342).

### 3.2. Cement–Dentin Interfaces

**Figure 4:**
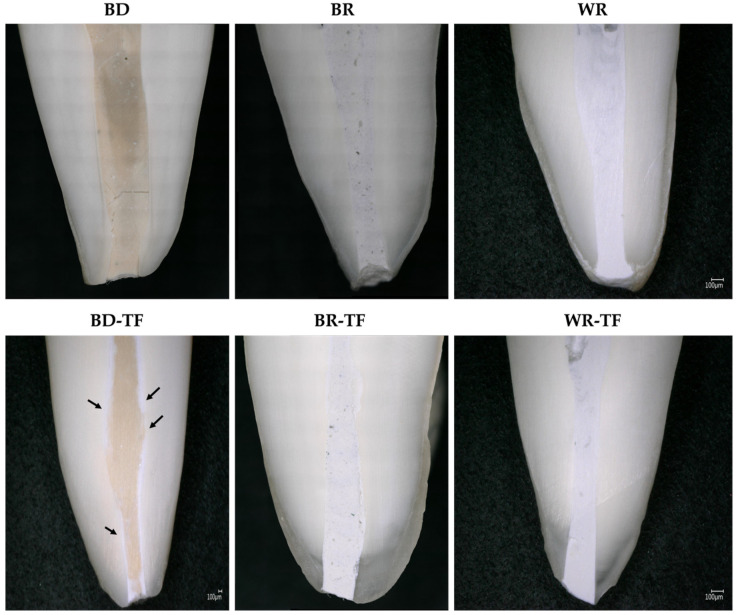
Digital micrographs showing the material–dentin interfaces in longitudinal sections teeth filled with the different cements and teeth filled with sealer–cements. Biodentine: BD; BR: MTA Biorep; Well-Root PT: WR; Biodentine + Total Fill: BD-TF; MTA Biorep + Total Fill: BR-TF; Well-Root PT+ Total Fill: WR-TF. Black arrows indicate the sealer layer at the dentin–material interface.

Good qualitative adaptations were obtained for all the groups (Figure 4). In the BD-TF micrograph, the distribution of sealer/cement was visible (black arrows) due to the different colors of the materials (Biodentine and sealer). The sealer was present on the dentinal walls and the Biodentine material was localized in the center of the root canal.

### 3.3. Cement–Dentin Interfaces (Tags)

**Figure 5:**
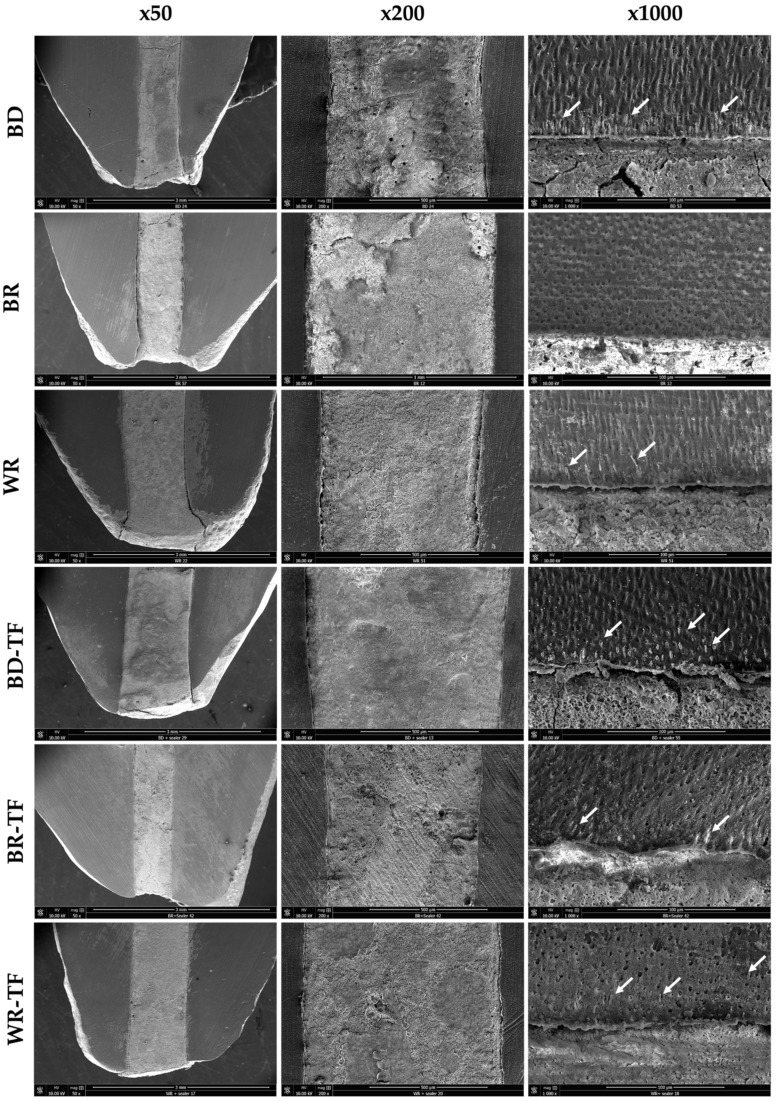
Scanning electron microscopy imaging (50×, 200× and 1000×) showing the material–dentin interfaces in longitudinal sections in Biodentine: BD; BR: MTA Biorep; Well-Root PT: WR; Biodentine + Total Fill: BD-TF; MTA Biorep + Total Fill: BR-TF; and Well-Root PT + Total Fill: WR-TF. White arrows indicate material infiltration into dentinal tubules.

**Figure 6:**
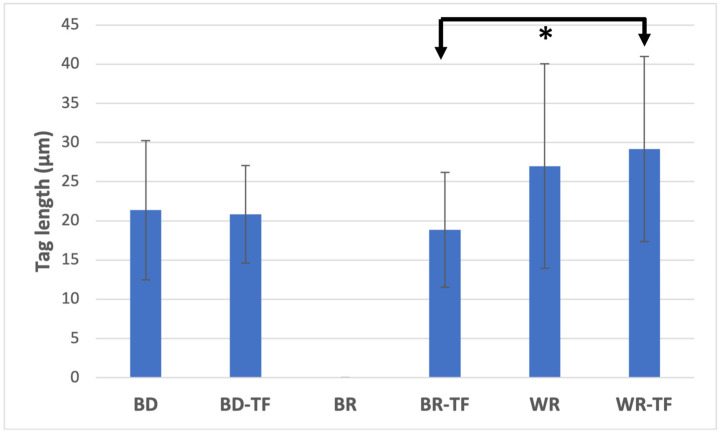
Means and standard deviation of material infiltration lengths into dentinal tubules. Biodentine: BD; BR: MTA Biorep; Well-Root PT: WR; Biodentine + Total Fill: BD-TF; MTA Biorep + Total Fill: BR-TF; Well-Root PT + Total Fill: WR-TF. (* *p* < 0.05).

All tags among the samples were detected at the coronal parts (>4 mm from the apex) of the apical plugs. Both cement groups without a sealer, G1 (BD) and G5 (WR), demonstrated material infiltrations, whilst G3 (BR) had no material infiltration into the dentinal tubules. No significant difference was found between the tag length of the WR and BD groups (*p* = 0.326). All the cement groups with sealer injection demonstrated material infiltrations into the dentinal tubules (Figure 5). WR-TF demonstrated significantly longer infiltrations (29.1 ± 12.1 µm) than BR-TF (18.8 ± 7.5 µm) (*p* = 0.047), whilst no significant differences were found between BD-TF (20.8 ± 6.3 µm) and BR-TF or WR-TF (*p* > 0.05) (Figure 6).

### 3.4. Cement–Sealer Interfaces

**Figure 7:**
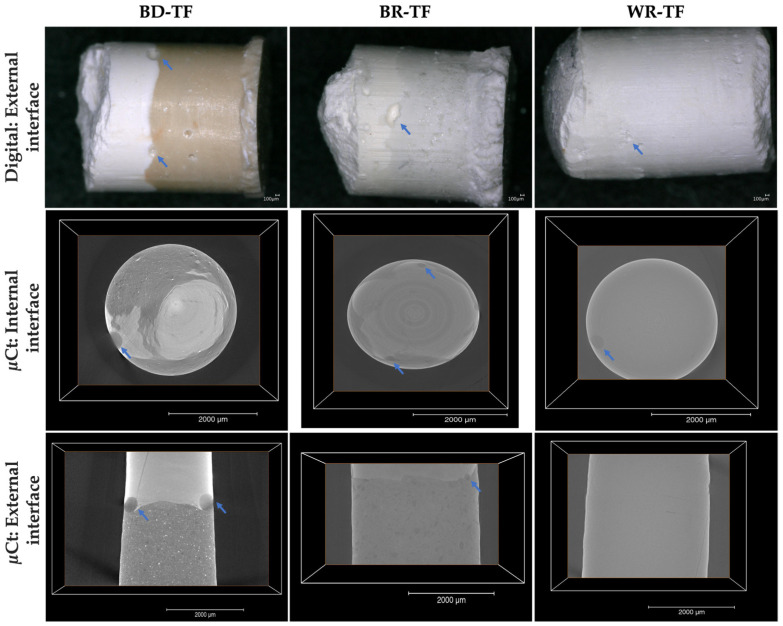
Digital micrographs (70× magnification) highlighting the cement–sealer external interfaces. Micro-computed-X-ray-tomography 2D slices showing the external and internal cement–sealer interfaces. Blue arrows indicate the presence of voids. Biodentine + Total Fill: BD-TF; MTA Biorep + Total Fill: BR-TF; Well-Root PT + Total Fill: WR-TF.

In all the groups, voids were observed at the cement–sealer interfaces (Figure 7, blue arrows; and Table 3). No statistical differences were found between the three tested groups (*p* > 0.05). The interfaces between BD and TF as well as between BR and TF were easily observed due to the difference in color between the tested materials. The WR-TF interface was difficult to image and contained few voids compared to BR-TF and BD-TF.

The results showed that all the voids were located on the external surfaces between the tested materials. The internal bonding interface among all the groups was well adapted with no detected voids. The WR-TF interface was also difficult to examine with the micro-computed X-ray tomography, as shown through the digital optical microscope (Figure 7). A significantly larger void volume was found for BD-TF compared to BR-TF and WR-TF (*p* = 0.001, *p* = 0.004, respectively) (Table 3), whilst no significant difference was found between WR-TF and BR-TF (*p* = 0.890). The difficulty in this investigation, as already shown for the digital observation, was still the possibility of detecting the interface between WR and TF.

## 4. Discussion

The success of root maturation after regenerative endodontic procedures in immature teeth with apical periodontitis and pulp necrosis is not always possible. In particular, immature roots with previously unsuccessful endodontic treatments fail to continue further root development in width and length [28]. Therefore, apexification could be considered as an alternative strategy to achieve apical barrier formation. The apical sealing of bioactive endodontic material as an apical plug-in an open apex model is an essential parameter for the clinical success of endodontic treatment. The hermetic seal of these materials plays an important role to avoid the reinfection of the root canal system by microorganisms [29]. Moreover, these bioactive endodontic materials have better biological and physicochemical properties than resin-based materials [30,31]. These cements have an alkaline pH and can release Ca^+2^ ions that could kill *bacteria* and enhance the remineralization process [15,19]. In addition, the shrinkage of resin materials during polymerization could create cohesive fractures and voids, whilst the calcium silicate material does not have this inconvenience [30]. Therefore, the purpose of the present in vitro study was to evaluate the marginal apical adaptation, material–dentin interface, and material infiltrations into the dentinal tubules for a premixed calcium aluminosilicate and two powder–liquid calcium silicate cements with or without the addition of a calcium silicate sealer before the obturation with cement.

The results showed that there was a statistically significant difference between the tested materials among material infiltrations into the dentinal tubules and the voids created between the cements and the sealer materials. Moreover, no significant differences were found between the tested materials among marginal apical adaptation. Therefore, the null hypothesis must be partially rejected.

A final irrigation protocol was performed by using 6% NaOCl and 17% EDTA to eliminate the smear layer and open the dentinal tubules. MapOne carrier was used to facilitate the delivery of endodontic materials into the canal system and the moistened floral sponge was used to simulate an apical barrier [26].

No significant difference was found between the marginal apical adaptation of the different tested groups. In addition, the use of a bioceramic sealer before obturation with cement did not ameliorate the marginal adaptation. In contrast, Tran et al. [5] compared the average gap size of cement obturation against the same cement associated with a sealer. Significantly fewer gaps are created when the cement is associated with its sealer. In the present study, there was no significant filling quality amelioration with the addition of the sealer. This could be related to the fact that the sealer used was shipped from another company other than that of the used cements, thus having different chemical compositions. Moreover, all the groups demonstrated well-adapted materials to the root canal and could be considered suitable for clinical application by orthograde obturation in an open apex model.

SEM observations demonstrated that some material infiltration occurred among all the tested groups except for the BR group at the coronal side of the apical plug. This finding could be related to the fact that the dentinal tubule diameter increases from the apical to the coronal thirds [32]. Larger tubule diameters could ameliorate the infiltration of materials into the dentinal structure. BR showed no tags, whilst BR-TF demonstrated some tags in the coronal side of the plug. This finding could be explained by the influence of the particle size and flowability of the tested materials [33,34]. The addition of a sealer that has a higher volume of finer and smaller particles, and higher flowability than MTA could ameliorate the penetration into the dentinal tubules [33,35,36]. BD demonstrated material infiltrations into the dentinal tubules at the coronal part of the apical plug. In accordance, Atmeh et al. [37] reported BD tags in the dentinal tubules. Moreover, WR demonstrated tags in the dentinal tubules; this could be due to the fact that the new premixed calcium silicate materials are fabricated from nanoparticles and some products included polymers [38]. In time and after four months, a calcium silicate material could create mineral depositions in the dentinal tubules that are responsible for killing the deep bacteria in the dentinal structure [39].

Longitudinal sections were observed by a digital optical microscope to evaluate the material–dentin interfaces as well as the distribution of the sealer and cement in the root canal system. The use of a digital optical microscope demonstrated in the BD-TF group that the sealer was placed on the dentinal walls and the cement was localized in the center of the obturation structure. Therefore, only the sealer was in contact with the dentinal walls and will react with the dentinal fluids and dentinal structure. BR-TF and WR-TF were not clearly observed due to the closed colors between the cements and the premixed sealer. But, normally, the sealer will be on the walls, as shown in the BD-TF group.

**Figure 8:**
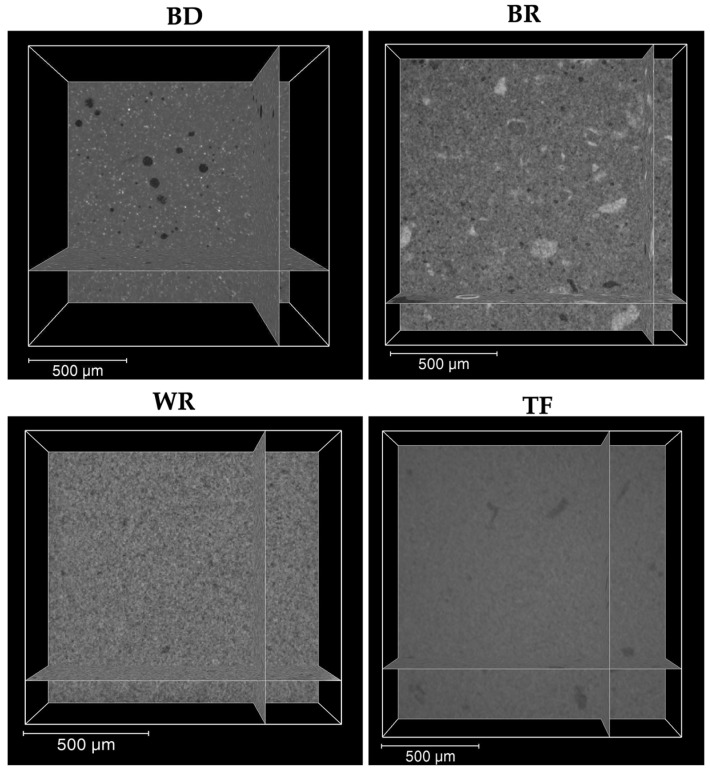
Micro-computed-X-ray-tomography 2D slices showing the internal structure of the three cements (Biodentine “BD”, MTA Biorep “BR” and Well-Root PT “WR”) and Total Fill sealer ‘’TF’’.

The interfaces between the different cements and the premixed sealer were firstly observed by a digital optical microscope. No significant differences were found between the tested groups. Moreover, the micro-computed X-ray tomography, as a more specific method, was used to investigate the internal and external structures of the materials in 3D. By using micro-computed X-ray tomography, BD-TF showed a larger number of voids compared to WR-TF and BR-TF. This could be due to the particle sizes, which are nanoscale for the premixed materials [39]. The obtained results (Figure 8) demonstrated similar structures of WR and TF, whilst BD and BR demonstrated different structures with qualitatively bigger particles. Moreover, the premixed form or the ready-to-use form do not need manual mixing or preparation before the application of the materials. Therefore, the ready-to-use product could reduce the errors that could alter the physicochemical properties of these materials [21]. In addition, Kharouf et al. [21] demonstrated that a premixed bioceramic could offer lower void percentages than a powder–liquid bioceramic. All the detected voids between the cements and the premixed sealer were localized at the external interface. Various studies [40,41] reported that the closed porosity within the materials could be considered as isolated infiltrated porosity that has almost no potential for bacterial migration. In addition, the coronal seal plays an important role in the success of open apex treatment because the microorganisms could penetrate through the root-filling materials along the dentinal walls in the area of open porosity, resulting in root-canal-system reinfection [30]. Consequently, the combination of a premixed cement and a premixed sealer in root canal treatment could offer lower voids between the two materials. In contrast, higher open pore percentages could be created by the combination of materials with different application modes, which play a role as a pathway for microorganisms. Moreover, further studies on the use of a combination of materials that contain the same/different chemical compositions should be performed. The findings of the present study support the clinical use of the novel premixed calcium aluminosilicate cement as root-end filling material. Moreover, dentists should respect the manufacturing instructions. In addition, as WR is commercialized as a capsule-form, ready-to-use product, there is no information about the expiry date after the opening of the capsule.

Several methods and techniques were proposed in order to ameliorate the filling ability of the endodontic materials and to minimize the void percentages. The use of ultrasonic activation could ameliorate the obturation quality and enhance the filling ability compared to manual compaction [42,43]. Moreover, the use of different applicators such as the MAP system could ameliorate the sealing ability of the apical plugs [44].

Further studies should be performed by using the non-invasive technique of tomography to investigate the void percentages and distinguish between the open and closed porosities. This technique could offer 3D imaging that could contribute to evaluating the quality of the obturation within the root canal system [45]. Moreover, a microleakage experiment should be carried out to evaluate the quality and stability of coronal and apical sealings with time. Marginal apical adaptation evaluation with longer storage periods for teeth obturated with bioceramic materials should be performed, as these materials are known for their high solubility [46]. In addition, further long-term studies in animal as well as clinical trials should be performed to evaluate the effects of these cements on the periapical tissues. While statistical significances were not detected for the marginal apical adaptation between the three tested materials, further study with a larger sample size should be performed in order to validate the findings. Moreover, only one technique of cement application and one technique of sealer injection were used in the present study. Further research should be performed to also compare different application techniques for the cement [47] and the sealer [48], which could change the filling ability.

## 5. Conclusions

Within the limitations of the present in vitro study, the three tested bioceramic cements demonstrated good marginal apical adaptation. The use of a sealer in the open apex model before the final obturation with cement does not improve the quality of the obturation, but it could increase penetration of the materials. The use of a sealer associated with cement could create external voids at the interfaces between the materials. The use of BD cement with a premixed sealer could increase the void percentages. The handling of a premixed bioceramic cement (WR) may be easier than would be the case with a powder–liquid cement (BD or BR).

## Figures and Tables

**Table 1:** Chemical composition, mixing method, and manufacturer of the used calcium-silicate-based cements [19,24,25].

Cement	Manufacturer	Lot	Mixing	Composition
Biodentine^TM^ “BD”	Septodont, Saint-Maur-des-Fossés, France	B25370	Powder: 1 capsule Liquid: 5 drops	Powder: tricalcium silicate; dicalcium silicate; calcium carbonate; zirconium dioxide; iron oxide Liquid: calcium chloride; water-soluble polymer
MTA Biorep “BR”	Itena Clinical, Paris, France	103308	Powder: 1 capsule Liquid: 4 drops	Powder: tricalcium silicate; dicalcium silicate; tricalcium aluminate; calcium oxide; calcium tungstate Liquid: water and plasticizer
Well-Root PT “WR”	Vericome, Gangwon-do, Republic of Korea	WT103100	Premixed	Calcium aluminosilicate compound; zirconium oxide; thickening agent
TotalFill^®^ BC Sealer™ “TF”	FKG, La Chaux-de-Fonds, Switzerland	21004SP	Premixed	Zirconium oxide, dicalcium silicate, tricalcium silicate, calcium phosphate monobasic, calcium hydroxide, filler, thickening agents

**Table 2:** Means and standard deviations of the assigned marginal apical adaptation scores. Biodentine: BD; BR: MTA Biorep; Well-Root PT: WR; Biodentine + Total Fill: BD-TF; MTA Biorep + Total Fill: BR-TF; Well-Root PT+ Total Fill: WR-TF.

Groups	G1	G2	G3	G4	G5	G6	*p*-Value
Scores	1.4 ± 0.6	1.1 ± 0.3	1.3 ± 0.4	1.0 ± 0.0	1.0 ± 0.0	1.2 ± 0.4	0.415

**Table 3:** Mean and standard deviations of the observed voids at the cement–sealer interfaces by using a digital optical microscope (µm^2^) and micro-computed X-ray tomography (µm^3^). Biodentine + Total Fill: BD-TF; MTA Biorep + Total Fill: BR-TF; Well-Root PT+ Total Fill: WR-TF. Different superscript letters (^a^, ^b^, and ^c^) indicate significant differences (*p* < 0.05).

Groups	BR-TF	WR-TF	BD-TF	Statistical Analysis
Digital microscope (µm^2^)	91,922 ± 35,706	10,386 ± 8347	54,796 ± 22,862	*p* > 0.05
Micro-computed X-ray tomography (µm^3^)	156.2 ± 63.66 ^a^	162.08 ± 127.54 ^b^	311.6 ± 34.23 ^c^	a–c: *p* = 0.001; b,c: *p* = 0.004

## Data Availability

The data presented in this study are available on request from the first author (raya.alrayes@damascusuniversity.edu.sy).
